# Supplementary figures and images for: Myco–Phycobiont Interactions within the “Ramalina farinacea Group”: A Geographical Survey over Europe and Macaronesia
Source: J Fungi (Basel). 2024 Mar 8;10(3):206. doi: 10.3390/jof10030206 (PMC10971535; doi:10.3390/jof10030206)

Scale 1:16.000.000  
Equidistant Conic Projection

Cartographic Service, University of León, Spain.  
(2001, April, 27)

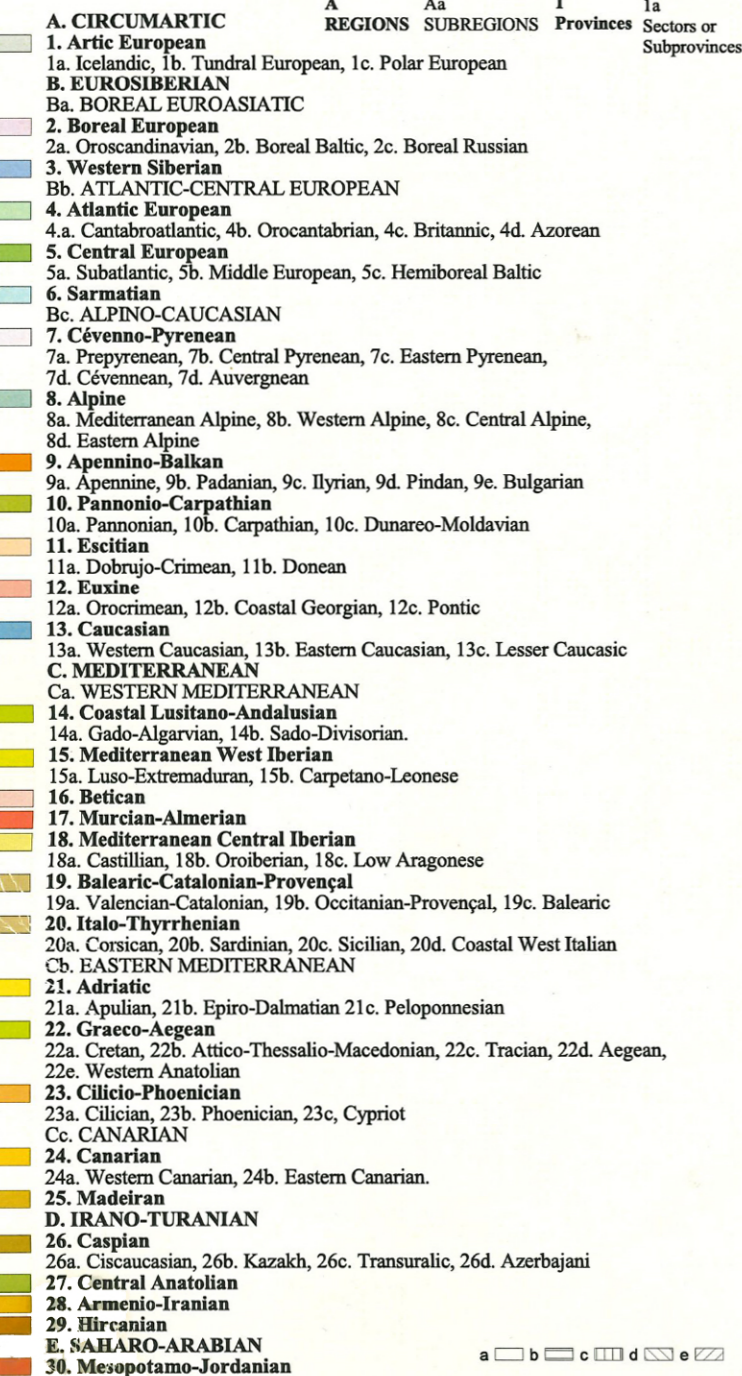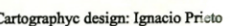

Supplement: Supplementary file 1 [file jof-10-00206-s001.zip › Figure S1.pdf]

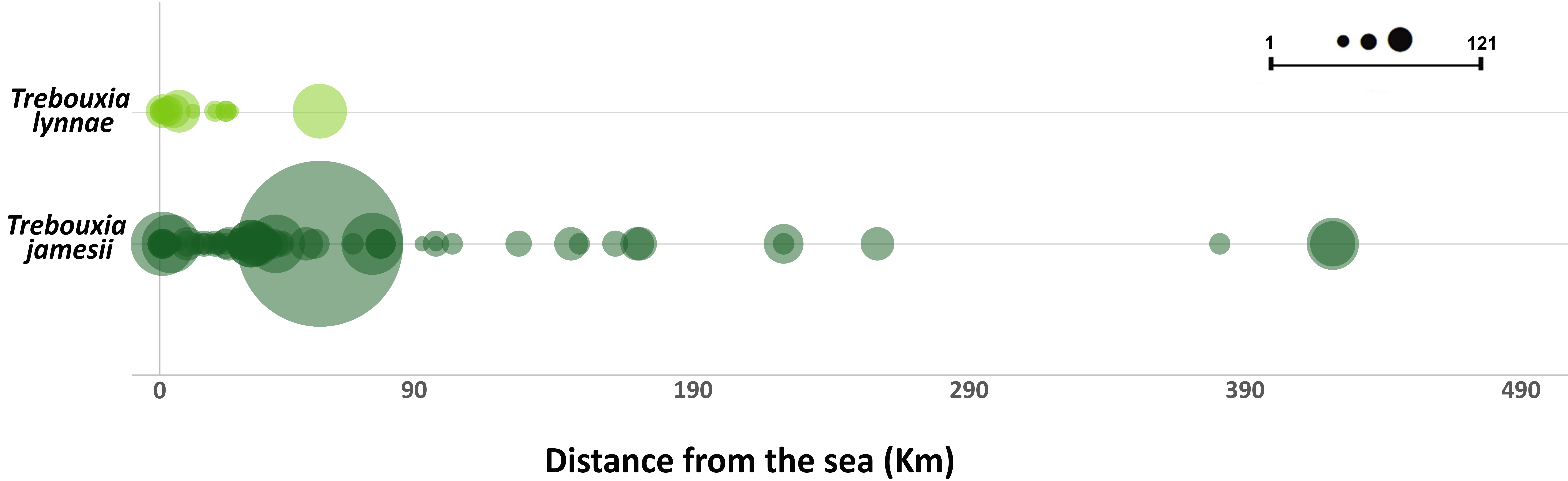

Supplement: Supplementary file 1 [file jof-10-00206-s001.zip › Figure S2.pdf]

*Trebouxia jamesii*

*Trebouxia lynnae*

Tree scale: 0.1

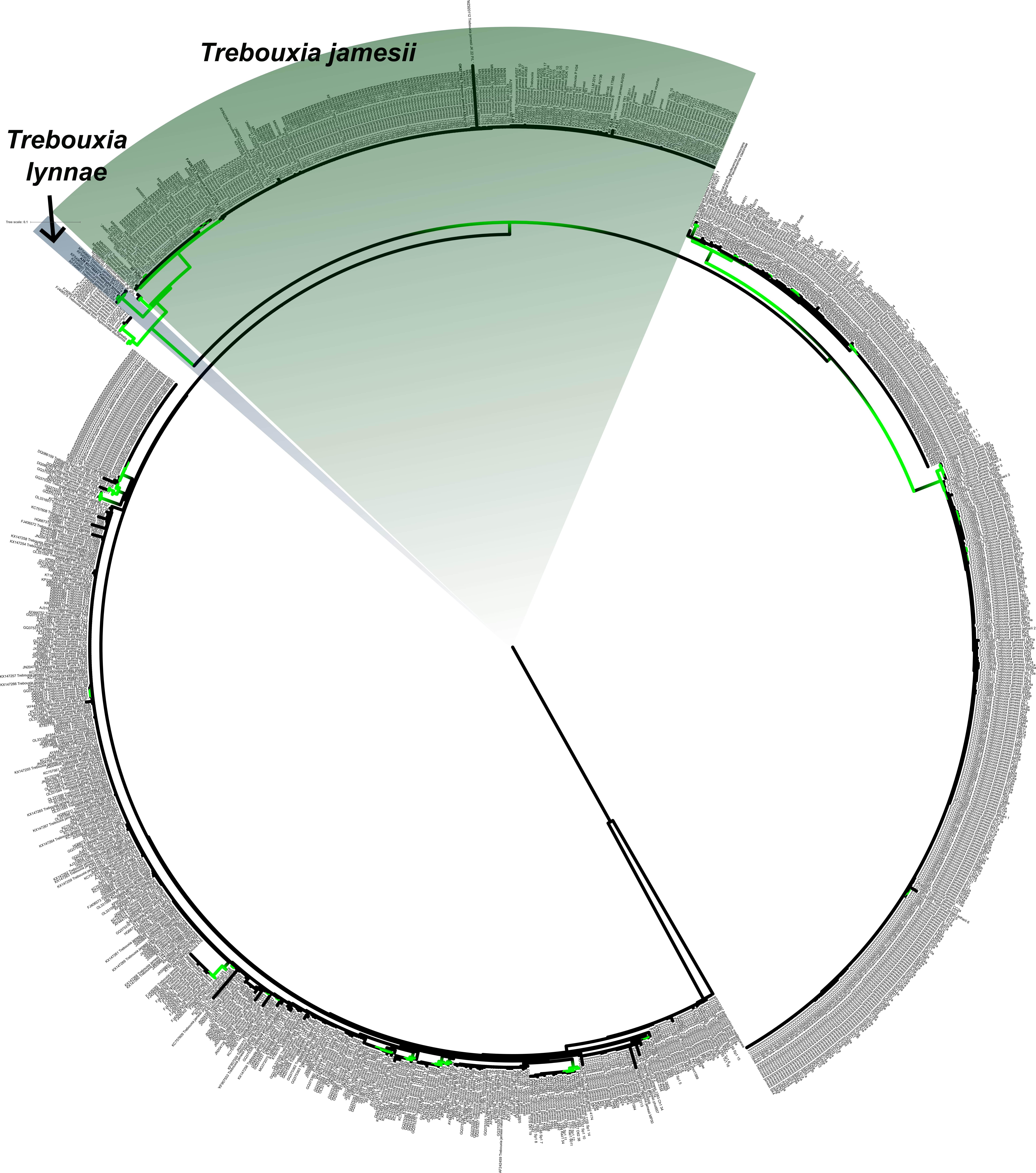

Supplement: Supplementary file 1 [file jof-10-00206-s001.zip › Figure S3.pdf]
